# Does the reform of the phased reduction of the pension insurance contribution rate benefit the labor income share of enterprises?

**DOI:** 10.3389/fpubh.2024.1473166

**Published:** 2025-01-17

**Authors:** Chengqiang Dou, Ying Liu

**Affiliations:** School of Public Administration, Shandong Technology and Business University, Yantai, China

**Keywords:** phased reduction, pension insurance contribution rate, capital intensity, labor income share, income distribution

## Abstract

**Introduction:**

This paper develops a theoretical model to clarify the mechanisms by which pension insurance contribution rate affects the labor income share of enterprises and conducts empirical validation.

**Methods:**

Utilizing a difference-in-differences approach, this paper analyzes data from China’s A-share listed companies from 2013 to 2018 to examine the impact and mechanism of the phased reduction of the pension insurance contribution rate on enterprise labor income share.

**Results and discussion:**

The phased reduction of the pension insurance contribution rate is beneficial for increasing enterprise labor income shares, with significant variations observed across regions and enterprise ownership types. The actual pension insurance contribution rate and capital intensity are two potential mechanisms through which this reform affects labor income share.

**Conclusion:**

In summary, we establish a causal link between the reform of the phased reduction of the pension insurance contribution rate and enterprise labor income share. This suggests that continuing the reform could increase labor income share, but it necessitates enhanced collection and management of pension insurance contributions, along with differentiated policies based on regional and enterprise ownership characteristics.

## 1 Introduction

**Figure 1:**
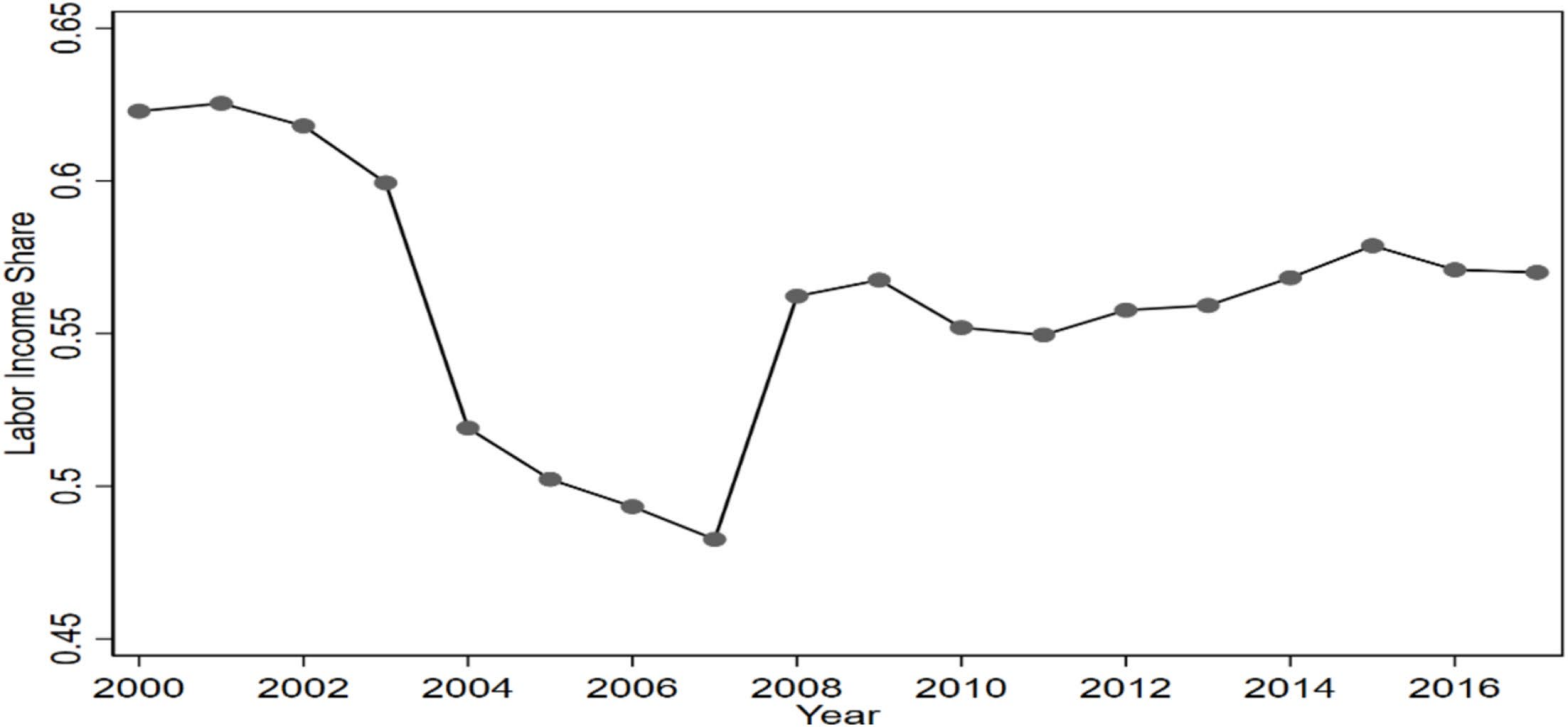
Trend of changes in China’s labor income share 2000–2017.

Over the past four decades of reform and opening-up, China’s economy has made great achievements. In 2022 China’s GDP reached 17.88 trillion US dollars, positioning it as the world’s second-largest economy. However, a concerning trend has emerged since 2000: China’s labor income share has been in a stage of persistent decline, with only a marginal recovery observed in 2007. Despite this, the labor income share remains at a relatively low (see Figure 1).^1^ The labor income share is an indicator of the proportion of labor compensation within national income, which is the portion of the fruits of economic development that is shared by workers (1). The sustained low level of labor income share suggests that the majority of workers have not been able to share the dividends of economic growth in a synchronized manner.

The labor income share is a key indicator of initial distribution fairness and crucial for narrowing income disparities. It is also a significant step toward achieving common prosperity. Research indicates that a decline in labor income share widens the income gap and disrupts social harmony and stability (2–5). Recognizing the pivotal role of the labor income share in alleviating income disparities, fostering social harmony and stability, and advancing common prosperity, scholars have engaged in extensive research to identify the determinants of labor income share. Extensive literature exists on factors such as technological progress (6, 7), financialisation (8, 9), taxation (10, 11), trade unions (12, 13), financing constraints (14), and foreign direct investment (15, 16). These studies have been approached from various perspectives and have yielded significant findings.

Recent studies have explored how minimum wage standards affect the labor income share (17–19). However, the impact of pension insurance contributions, a major component of labor costs, on labor income share has been less studied. The theory of tax burden shifting suggests that businesses struggle to pass on their full pension insurance costs, leading to increased labor costs when these contributions rise. The factor substitution theory indicates that higher labor costs prompt businesses to increase capital investment at the expense of labor, reducing labor income share. Therefore, it is necessary to investigate the effects of pension insurance contributions on the labor income share.

In 2016, China initiated a reform to phased reduce pension insurance contribution rate, offering an opportunity to study the effect of these rate on labor income share. The Ministry of Human Resources and Social Security and the Ministry of Finance issued a joint circular that permitted provinces to decrease the employer contribution rate for basic pension insurance to 20% or 19% from May 2016 for 2 years, depending on local circumstances. This paper uses the reform as a “quasi-natural experiment” and applies a difference-in-differences model to evaluate its impact on enterprise labor income share and related mechanisms, using data from China’s A-share listed companies from 2013 to 2018.

In contrast to prior studies, this paper’s innovation is primarily manifested in the following respects: (1) This paper analyzes how the reform of the phased reduction of the pension insurance contribution rate affects labor income share from the perspective of labor cost changes, offering valuable insights for China’s efforts to raise labor income share and achieve common prosperity amidst pension system reforms. (2) Employing the phased reduction reform as a “quasi natural experiment,” the paper uses the difference-in-differences method to establish a causal link between pension insurance contribution rate and labor income share, addressing endogeneity and bolstering the robustness of the results. (3) The paper develops a theoretical model to explain the mechanism of how pension insurance contribution rates affect labor income share and confirms this mechanism empirically, expanding research on the economic effects of pension insurance and the factors influencing labor income share.

## 2 Institutional background

Although China’s pension insurance system has undergone continuous adjustments and enhancements since its inception, several pressing issues persist, notably the high pension insurance contribution rate.^2^ Prior to 2016, enterprise pension insurance contribution rate varied significantly across provinces: 22% in Heilongjiang, 21% in Shanghai, 18% in Shandong and Fujian, 14% in Guangdong and Zhejiang, and 20% in the other provinces^3^. Such elevated rate exacerbate the burden on enterprises, which hinders the promotion of corporate vitality.

In April 2016, the Ministry of Human Resources and Social Security and the Ministry of Finance jointly promulgated the Notice on Phased Reduction of Social Insurance Contribution Rate. This policy aimed to alleviate enterprise costs, enhance corporate vitality, and further refine the pension insurance system. It mandated a gradual reduction in the employer contribution rate for basic pension insurance: provinces with a rate exceeding 20% were to adjust it down to 20%, while those with a 20% rate and a sufficient fund balance at the end of 2015 to cover pensions for over 9 months could reduce their rate to 19%.^4^ In response, provinces actively implemented these reforms, leading to a sequential reduction in pension insurance contribution rate. For instance, Heilongjiang reduced its rate from 22 to 20%, Shanghai from 21 to 20%, and 18 provinces including Beijing, Tianjin, and Chongqing from 20 to 19%.^5^ However, 11 provinces like Guangdong and Hebei maintained their rate.^6^ Post-reform, most provinces have a 19% rate, which, despite the reduction, remains significantly higher than those in developed countries, such as the United States at 6.2%, Japan at 8.9%, and the United Kingdom at 13.8%.^7^

Theoretically, a reduction in the pension insurance contribution rate can reduce the enterprises’ pension insurance contribution, which is an important factor affecting the labor income share. Therefore, the reform of phased reduction of pension insurance contribution rate provides a good opportunity for this paper to identify the impact of the pension insurance contribution rate on the labor income share of enterprises.

## 3 Theoretical model

This paper examines the theoretical implications of pension insurance contribution rate on the labor income share by developing a theoretical model that incorporates such rate. Building upon the theoretical frameworks of Wan and Wei (18), Du et al. (17) and Yin et al. (20), the model introduces relevant parameters. It is anchored in the constant returns to scale assumption of the CES production function. Through this framework, the paper seeks to elucidate the mechanisms through which pension insurance contribution rate affect the labor income share and to propose research hypotheses for empirical validation.

In a perfectly competitive market, a typical firm i utilizes two input factors (labor factor $L_{i}$ and capital factor $K_{i}$ ). To streamline the analytical process, the production function $Y_{i}$ is modeled as a constant elasticity of substitution (CES) function. The specific formulation of the production function is presented as follows:


(1) $$Y_{i}=A_{i}{\left[\alpha {K_{i}}^{\frac{\sigma -1}{\sigma }}+\left(1-\alpha \right){L_{i}}^{\frac{\sigma -1}{\sigma }}\right]}^{\frac{\sigma }{\sigma -1}}$$

Where $Y_{i}$ is the firm’s output level, $A_{i}\in \left(0,\infty \right)$ which denotes the firm’s production efficiency, reflecting the level of technological progress; $\alpha \in \left(0,1\right)$ , which denotes the factor intensity parameter, and the substitution parameter $\sigma \in \left[0,\infty \right]$ , which denotes the elasticity of substitution between capital and labor factors.

A typical firm achieves its profit-maximizing production objective by choosing the optimal capital $K_{i}$ and labor $L_{i}$ . Consequently, the firm’s profit maximization problem can be formulated as follows:


(2) $$\max_{K_{i},L_{i}}\pi_{i}=P_{i}Y_{i}-\left(1+\theta \tau_{i}\left(\mu \right)\right)w_{i}L_{i}-r_{i}K_{i}$$

Where the price per unit of product is $P_{i}$ . The rental rate for the standard capital element is denoted by $r_{i}$ , while $w_{i}$ signifies the wage rate for standard labor input, excluding social insurance contributions. The pension insurance contribution rate is represented by *μ* and τ denotes the actual pension insurance contribution rate. The parameter θ is defined as $\frac{1}{\rho }$ , where ρ is the proportion of pension insurance contributions to total social insurance contributions. Zheng (21)observed that the proportion of pension insurance contributions to all social insurance contributions in each region is generally stable at 65%. This stability allows us to reasonably infer a consistent proportional relationship between the actual pension insurance contribution rate and the actual social insurance contribution rate. The term $\theta \tau_{i}\left(\mu \right)$ signifies the actual social insurance contribution rate.

Substitute Equation 1 into Equation 2, then solve the problem of maximizing its profit, and finally perform mathematical transformation to obtain Equation 3.


(3) $$\frac{r_{i}}{\left(1+\theta \tau_{i}\left(\mu \right)\right)w_{i}}=\frac{\alpha }{1-\alpha }{\left(\frac{L_{i}}{K_{i}}\right)}^{\frac{1}{\sigma }}$$

In equilibrium, a firm’s output is precisely equal to the sum of the incomes derived from the capital and labor factors.


(4) $$P_{i}Y_{i}=\left(1+\theta \tau_{i}\left(\mu \right)\right)w_{i}L_{i}+r_{i}K_{i}$$

Define the labor income share $LS_{i}$ as the ratio of labor factor income $\left(1+\theta \tau_{i}\left(\mu \right)\right)w_{i}L_{i}$ to total factor incomes $P_{i}Y_{i}$ . That is:


(5) $$LS_{i}=\frac{\left(1+\theta \tau_{i}\left(\mu \right)\right)w_{i}L_{i}}{P_{i}Y_{i}}$$

By substituting Equation 4 into Equation 5 and collapsing gives:


(6) $$LS_{i}=\frac{\left(1+\theta \tau_{i}\left(\mu \right)\right)w_{i}L_{i}}{\left(1+\theta \tau_{i}\left(\mu \right)\right)w_{i}L_{i}+r_{i}K_{i}}=\frac{1}{1+\frac{r_{i}}{\left(1+\theta \tau_{i}\left(\mu \right)\right)w_{i}}\times \frac{K_{i}}{L_{i}}}$$

Define capital intensity $k_{i}$ as the ratio of capital factor input to labor factor input, such that $k_{i}=\frac{K_{i}}{L_{i}}$ .

An escalation in the actual pension insurance contribution rate leads to an increase in the relative price of capital versus labor, thereby inducing factor substitution effects. Consequently, this alters the firm’s factor input decision-making. Thus, the degree of capital intensity $k_{i}$ serves as the response function to the actual pension insurance contribution rate.


(7) $$k_{i}\left(\tau_{i}\right)=\frac{K_{i}\left(\tau_{i}\right)}{L_{i}\left(\tau_{i}\right)}$$

By incorporating Equations 7, 3 into Equation 6 and combining them, we derive:


(8) $$LS_{i}\left(\mu \right)=\frac{1}{1+\frac{\alpha }{1-\alpha }k_{i}{\left(\tau_{i}\right)}^{\frac{\sigma -1}{\sigma }}}=\frac{1}{1+\frac{\alpha }{1-\alpha }{\left[k_{i}\left(\tau_{i}\right)\right]}^{\frac{\sigma -1}{\sigma }}}$$

Equation 8 reveals t that the labor income share in equilibrium for firms is directly influenced by the elasticity of substitution σ between capital and labor factors, the capital intensity $k_{i}$ , and the actual pension insurance contribution rate $\tau_{i}$ .

Equation 8 explicitly delineates the relationship between the pension insurance contribution rate and the labor income share. Consequently, the partial derivative of the labor income share *LS* with respect to the pension insurance contribution rate μ is derived from Equation 8:


(9) $$\begin{array}{l} \frac{\partial LS_{i}\left(\tau_{i}\right)}{\partial \mu }=\frac{\partial LS_{i}\left(\tau_{i}\right)}{\partial k_{i}\left(\tau \right)}\times \frac{\partial k_{i}\left(\tau_{i}\right)}{\partial \tau_{i}}\times \frac{\partial \tau_{i}\left(\mu \right)}{\partial \mu }\\ =\frac{\frac{\alpha \left(1-\sigma \right)}{\left(1-\alpha \right)\sigma }k_{i}{\left(\tau \right)}^{\frac{-1}{\sigma }}}{{\left(1+\frac{\alpha }{1-\alpha }k_{i}{\left(\tau \right)}^{\frac{\sigma -1}{\sigma }}\right)}^{2}}\times \frac{\partial k_{i}\left(\tau \right)}{\partial \tau_{i}}\times \frac{\partial \tau_{i}\left(\mu \right)}{\partial \mu } \end{array}$$

From Equation 9 illustrates that, given, a specific elasticity of substitution between capital and labor factors, the influence of the pension insurance contribution rate on the labor income share is directed by two factors: the sensitivity of capital intensity to the actual pension insurance contribution rate $\frac{\partial k_{i}\left(\tau_{i}\right)}{\partial \tau_{i}}$ and the responsiveness of the actual pension insurance contribution rate to the nominal rate $\frac{\partial \tau_{i}\left(\mu \right)}{\partial \mu }$ . This indicates that the firm’s capital intensity $k_{i}$ and the actual contribution rate of pension insurance $\tau_{i}$ constitute the economic mechanisms that mediate how changes in the pension insurance contribution rate affect the labor income share.

First, this paper initially posits that the elasticity of substitution σ between capital and labor factors exceeds 1, indicating a substitution relationship between capital and labor factors.^8^ Empirical evidence from studies on Chinese industrial data supports this assertion, revealing a substitution effect between capital and labor (18, 22, 23).

Secondly, this paper posits that the capital intensity is responsive to the actual pension insurance contribution rate, with $\frac{\partial k_{i}\left(\tau_{i}\right)}{\partial \tau_{i}}>0.$ ^9^

Given that pension insurance contributions constitute a significant element of labor costs, an augmentation in the actual pension insurance contribution rate elevates these costs. Consequently, the relative price of labor to capital increases, fostering a substitution effect where labor is replaced by capital. This necessitates an adjustment in firms’ factor input decisions, specifically an augmentation of capital input and a reduction in labor input, thereby increasing capital intensity. Hence, it is concluded that an escalation in the actual pension insurance contribution rate will lead to an increase in the capital intensity of firms.

This paper ultimately contends that the actual pension insurance contribution rate is positively responsive to changes in the statutory pension insurance contribution rate, denoted as $\frac{\partial \tau_{i}\left(\mu \right)}{\partial \mu }>0$ . Analyzing data from listed companies between 2007 and 2018, Cheng et al. (27) discovered that the reform aimed at the phased reduction of pension insurance contribution rate effectively lowered the actual pension insurance contribution rate for enterprises. This finding indicates a positive correlation between the statutory pension insurance contribution rate and the actual rate faced by enterprises.

Following the aforementioned analyses, this paper proposes the initial research hypothesis for examination:

Research Hypothesis 1: The reform of phased reduction of the pension insurance contribution rate leads to a decrease in the pension insurance contribution rate, consequently lowering the actual contribution rate of enterprises’ pension insurance, and ultimately leading to a reduction in the capital intensity of enterprises.

Research Hypothesis 1 posits that there is a positive correlation between the actual pension insurance contribution rate and the statutory pension insurance contribution rate, as well as between capital intensity and the pension insurance contribution rate. This is mathematically represented as.

Research hypothesis 1 implies that there is a positive relationship between the actual pension insurance contribution rate and the pension insurance contribution rate, as well as between capital intensity and the pension insurance contribution rate. This is mathematically represented as $\frac{\partial \tau_{i}\left(\mu \right)}{\partial \mu }>0\;\mathrm{and}\;\frac{\partial k_{i}\left(\tau_{i}\right)}{\partial \tau_{i}}>0$ .

Given that the elasticity of substitution σ>1 between the capital and labor factors, the sign of Equation 11 remains consistently negative, indicating that the labor income share is a decreasing function of the pension insurance contribution rate, assuming Research Hypothesis 1 is valid. Consequently, this paper proposes the second research hypothesis for evaluation.

Research hypothesis 2: The reductions in the pension insurance contribution rate, induced by the reform of phased reduction, are conducive to an increase in the enterprises’ labor income share.

Synthesizing the aforementioned analysis, this paper can deduce the theoretical mechanism by which the reform of phased reduction of pension insurance contribution rate is conducive to the improvement of enterprises’ labor income share as follows:

Firstly, the reform of phased reduction of pension insurance contribution rate results in a decrease in the statutory pension insurance contribution rate, consequently leading to a reduction in the actual pension insurance contribution rate and contributions for enterprises. Secondly, as pension insurance contributions constitute a significant element of labor costs, this reform effectively raises the relative price of capital for enterprises. This increase in the relative price of capital subsequently induces a decrease in capital intensity, which ultimately fosters an increase in the labor income share of enterprises. The paper subsequently uses relevant data to empirically test the research hypothesis.

## 4 Research design

### 4.1 Data

The time frame in this paper spans from 2013 to 2018. This period was selected to minimize the effects of the 2012 transition from business tax to value-added tax and the 2019 《Comprehensive Plan for Reducing Social Insurance Premiums》 on the labor income share of enterprises. The primary data, encompassing basic and financial information of A-share listed companies in the Shanghai and Shenzhen stock markets, are extracted from the RISI Financial Database and the Cathay Pacific China Economic and Financial Database. Complementary provincial-level data are derived from the China Statistical Yearbook for each respective year. Information regarding the reform of the phased reduction of pension insurance contribution rate is primarily gathered from the official websites of provincial human resources and social security departments, as well as people’s governments across various provinces, with additional data obtained through Baidu searches.

This paper selects the sample of listed companies based on specific exclusion criteria: (1) companies in the financial and insurance sectors are excluded; (2) companies with an ST or *ST designation are excluded; (3) companies lacking essential data for key variables are excluded; (4) companies listed after 2016 are excluded from the sample; (5) companies with a labor income share outside the range of 0 to 1 are excluded. To mitigate the influence of outliers, the paper trims continuous variables at the 1st and 99th percentiles for each firm. After applying these filters, the study includes a total of 7,833 samples from 1,574 distinct listed companies.

### 4.2 Model design

This paper leverages the quasi-natural experiment afforded by the phased reduction of pension insurance contribution rate, initiated by Chinese provinces in 2016, to construct a difference-in-differences model. This model is employed to assess the reform’s impact on the labor income share of enterprises.


(10) $$\begin{array}{l} LS_{\mathrm{pit}}=\beta_{0}+\beta_{1}Tt_{\mathrm{pi}}\times Pt_{\mathrm{pt}}+X_{\mathrm{pit}}\times \lambda \\ \;+Z_{\mathrm{pt}}\times \theta +\mathrm{Fir}m_{i}+Year_{t}+\in_{\mathrm{pit}} \end{array}$$

where the subscripts p,iandt represent province, firm, and year, respectively. The dependent variable $LS_{\mathrm{pit}}$ represents the labor income share of the *i*th firm in province *p* during year *t*;

The dummy variable $Tt_{\mathrm{pi}}$ indicates firm grouping, where a value of 1 signifies the treatment group and 0 signifies the control group. The time grouping dummy variable $Pt_{\mathrm{pt}}$ is defined, taking a value of 0 for the years 2013–2015 and 1 for 2016–2018. The estimated coefficient $\beta_{1}$ quantifies the reform’s impact on factor income distribution within a difference-in-differences framework, with a positive sign anticipated.

The vector $X_{\mathrm{pit}}$ encompasses a set of firm-level control variables that potentially influence the labor income share. The vector $Z_{\mathrm{pt}}$ represents provincial-level control variables. The term $\mathrm{Fir}m_{i}$ signifies firm fixed effects, $Year_{t}$ signifies time fixed effects, and $\in_{\mathrm{pit}}$ denotes the random error term.

### 4.3 Variable definition

#### 4.3.1 Dependent variable

The dependent variable in this paper is the labor income share (*LS*), which is defined as the proportion of labor compensation in the firm’s value added. Following Wang and Huang (16), labor compensation in this paper is operationalized as the total incidence of employee compensation paid, encompassing both monetary and non-monetary forms of compensation provided to employees during and after their employment. This comprehensive measure better captures the total labor compensation within the enterprise. The value added of an enterprise is calculated as the sum of labor compensation, operating profit, and the depreciation of fixed assets. Operating profit is derived by subtracting operating costs from the enterprise’s operating income.

#### 4.3.2 Independent variable

The independent variable is the reform of phased reduction of pension insurance contribution rate, denoted as Tt×Pt , which represents the interaction term between the firm grouping dummy variable ( Tt ) and the time grouping dummy variable ( Pt) . Firms are categorized as being in the treatment group if they are located in a province that has implemented the reform, with Tt takes the value of 1; Otherwise, they are in the control group with Tt assigned a value of 0. The provinces where the treatment group and control group are located are shown in Table 1. The variable Pt is set to 0 for the period prior to the reform’s implementation and to 1 for the years during and after the implementation. Given that the reform commenced in 2016, Pt equals 1 for years 2016 and beyond, and 0 for earlier years.

**Table 1:** Provinces where the treatment group and control group are located.

Group name	Province where it is located
Treatment group	Heilongjiang, Shanghai, Beijing, Tianjin, Chongqing, Sichuan, Anhui, Jiangxi, Xinjiang, Shanxi, Henan, Hubei, Guangxi, Guizhou, Hunan, Gansu, Ningxia, Jiangsu, Hainan and Yunnan.
Control group	Guangdong, Hebei, Fujian, Zhejiang, Liaoning, Jilin, Qinghai, Shandong, Inner Mongolia, and Shaanxi. Tibet was excluded because no firms were located in Tibet in the processed sample.

Tibet was excluded because no firms were located in Tibet in the processed sample.

#### 4.3.3 Control variables

Referring to Xu et al. (24) and Zhu and Jiang (25), the control variables specifically encompass: (1) Debt ratio (*Lev*), calculated as the proportion of the enterprise’s total liabilities to its total assets; (2) Enterprise growth (Growth), measured as the ratio of the current year’s operating revenue minus the previous year’s operating revenue to the previous year’s operating revenue; (3) Bank borrowing (Bank), quantified as the proportion of the sum of short-term and long-term borrowings to the enterprise’s total assets; (4) Profitability (Roa), expressed as the ratio of net profit to total assets. (5) Management shareholding (Mshare), calculated as the ratio of the number of shares held by management to the total number of shares issued by the enterprise. (6) Board size (Board), defined as the natural logarithm of board size. (7) Balances (Balances), defined as the ratio of the sum of the shareholdings of the second to tenth largest shareholders to the shareholding of the first largest shareholder; (8) Age of the firm (*Age*), defined as the natural logarithm of the number of years the firm has been in existence; (9) Tobin’s Q (*Tq*), defined as the ratio of the sum of the market value of equity, the book value of debt, and the book value of the firm’s total assets; (10) Cash substitutes (*Liqui*), defined as the ratio of the difference between working capital, money funds, and the total assets of the firm; (11) The level of economic development (*Pgdp*), defined as the provincial GDP per capita; (12) The average wage of employees (*Awage*), defined as the natural logarithm of the average wage of employees in the province.

## 5 Empirical results

### 5.1 Descriptive statistics

The descriptive statistics results of the primary variables are presented in Table 2. The table indicates that the labor income share of enterprises has a sample mean of 0.2871, with a standard deviation of 0.1244, a minimum value of 0.0575, a median of 0.2742, and a maximum of 0.7208. These statistics suggest that there is relatively modest variation in the labor income share among enterprises, and the overall level of labor income share remains comparatively low. Additionally, the sample mean of the dummy variable (*Tt*), which represents the enterprise grouping, is 0.5524. This figure signifies that 55.24% of the enterprises in the sample are situated in provinces that have adopted the reform for the phased reduction of pension insurance contribution rate.

**Table 2:** Descriptive statistics of main variables.

Variables	*N*	Mean	Standard Deviation	Minimum	Median	Maximum
LS	7,833	0.2871	0.1244	0.0575	0.2742	0.7208
Tt	7,833	0.5524	0.4973	0.0000	1.0000	1.0000
Pt	7,833	0.4561	0.4981	0.0000	0.0000	1.0000
Lev	7,833	0.4911	0.1942	0.0770	0.4948	0.9085
Growth	7,833	0.1841	0.4020	−0.4952	0.1130	2.4509
Bank	7,833	0.1813	0.1287	0.0000	0.1687	0.5494
Roa	7,833	0.0296	0.0509	−0.2158	0.0278	0.1637
Mshare	7,833	0.1026	0.1715	0.0000	0.0016	0.6387
Tq	7,833	2.2694	1.5355	0.8572	1.7518	9.1973
Age	7,833	2.8745	0.3184	1.3863	2.8904	3.9318
Balances	7,833	0.8987	0.7624	0.0418	0.6833	3.6945
Board	7,833	2.1502	0.1965	1.6094	2.1972	2.7081
Liqui	7,833	0.0051	0.1974	−0.4978	0.0053	0.4701
Pgdp	7,833	11.0884	0.4031	10.0498	11.1199	11.8509
Awage	7,833	11.1197	0.2914	10.5532	11.0849	11.8898

### 5.2 Benchmark regression results

The benchmark regression analysis examining the impact of the reform of phased reduction of pension insurance contribution rate on the labor income share of enterprises is presented in Table 3. Column (1) of Table 3 includes province-level control variables, time fixed effects, and firm fixed effects, revealing an estimated coefficient for Tt×Pt is 0.0102, which is significantly positive at the 1% level. This suggests that the reform has a substantial positive effect on the labor income share of enterprises. Column (2) incorporates firm-level control variables, time fixed effects, and firm fixed effects, yielding an estimated coefficient for Tt×Pt is 0.0058, significantly positive at the 5% level. These the regression results indicate that the reform of phased reduction of pension insurance contribution rate has led to approximate increase of 2.02%^10^ in the labor income share of enterprises. Column (3) adds both firm-level and province-level control variables, while also accounting for time fixed effects and firm fixed effects, which not only addresses potential omitted variable bias but also enhances the precision of the regression outcomes. This paper adopts the regression results from column (3) of Table 3 as the benchmark for interpretation. Therefore, this paper adopts the regression results from column (3) of Table 3 as the benchmark for interpretation. The estimated coefficient of Tt×Pt is 0.0072 and is significantly positive at the 1% level. This suggests that the reform of the phased reduction of pension insurance contribution rate has resulted in an approximate 2.51%^11^increase in the labor income share of enterprises in the provinces where the reform was implemented. Collectively, these benchmark regression findings indicate that the reform is advantageous to the labor income share of enterprises. Consequently, Research Hypothesis 2 is supported.

**Table 3:** Benchmark regression results.

Variable	(1)	(2)	(3)
*Tt × Pt*	0.0102^***^ (0.0032)	0.0058^**^ (0.0025)	0.0072^***^ (0.0027)
Lev		−0.0433^**^ (0.0175)	−0.0429^**^ (0.0175)
Growth		−0.0287^***^ (0.0025)	−0.0287^***^ (0.0025)
Bank		−0.0137 (0.0198)	−0.0136 (0.0198)
Roa		−0.7901^***^ (0.0574)	−0.7899^***^ (0.0575)
Mshare		−0.0171 (0.0148)	−0.0171 (0.0148)
Tq		0.0009 (0.0010)	0.0008 (0.0010)
Age		−0.0648^***^ (0.0229)	−0.0629^***^ (0.0229)
Balances		−0.0095^***^ (0.0025)	−0.0093^***^ (0.0025)
Board		0.0108 (0.0087)	0.0108 (0.0087)
Liqui		−0.0191 (0.0133)	−0.0191 (0.0133)
Pgdp	−0.0078 (0.0246)		−0.0219 (0.0185)
Awage	0.0932^*^ (0.0515)		0.0528 (0.0465)
Constant	−0.6649 (0.5969)	0.5094^***^ (0.0698)	0.1593 (0.4905)
Firm/Year	Yes	Yes	Yes
*R^2^*_adjust	0.7341	0.8101	0.8101
*N*	7,833	7,833	7,833

Standard errors clustered to province-year are in parentheses. Subsequent tables are identical. The symbols ***, **, and * respectively denote *p* < 0.01, *p* < 0.05, and *p* < 0.1.

The estimation results for the control variables are largely consistent with expectations. The coefficient for *Lev* is significantly negative at the 5% level, suggesting that higher leverage is associated with a lower labor income share of enterprises, a finding that aligns with Wang and Huang (16). The estimated coefficient for *Roa* is −0.7899 and significant at the 1% level, indicating that higher profitability is associated with a lower labor income share, possibly because the distribution of factor income favors capital owners. The coefficient for *Age* is significantly negative, implying that the older the firm, the lower its labor income share instead, possibly due to a decrease in labor bargaining power as firm age increases, which hinders the growth of the labor income share and is consistent with Wan and Wei (18). The coefficient for *Balances* is significantly negative, indicating that enterprises with higher equity balances have a lower labor income share. The remaining control variables, which are not further analyzed, are insignificant and have limited practical relevance.

To investigate the differential impact of the reform of phased reduction of pension insurance contribution rate on the labor income share of different types of labor force, this paper divides the labor income share of enterprises into the executive labor income share and the employee labor income share bifurcated based on labor force type: the executive labor income share is calculated as the ratio of executive compensation to the enterprise’s value added, while the employee labor income share is determined as the ratio of the residual labor compensation, excluding executive compensation, to the enterprise’s value added. The detailed regression outcomes for these segments are displayed in Table 4.

Table 4 shows that the coefficient of the impact of the reform of phased reduction of pension insurance contribution rate on the employee labor income share exceeds that of the executive labor income share, with both coefficients being significantly positive at the 1% level. This suggests that the reform’s influence on the labor income share of enterprises is primarily manifested in the enhancement of the employee labor income share, rather than the executive labor income share. Potential reasons include the high level and diversity of income sources for enterprise executives, which render them less responsive to changes in pension insurance contribution rate. Conversely, ordinary employees, with lower income levels and a singular source of labor income, exhibit lower income elasticity and greater sensitivity to such rate changes. Furthermore, ordinary employees possess lower skill levels and weaker bargaining power. When fluctuations in pension insurance contribution rate lead to labor cost adjustments, enterprises can readily hire or release ordinary employees. Consequently, the phased reduction in pension insurance contribution rate has a more pronounced effect on the employee labor income share within enterprises.

**Table 4:** Executives labor income share and employees labor income share.

Variable	(1)	(2)
	Executives labor income share	Employees labor income share
*Tt × Pt*	0.0004^***^ (0.0001)	0.0070^***^ (0.0027)
Constant	0.0157 (0.0163)	0.1430 (0.4825)
Controls	Yes	Yes
Firm/Year	Yes	Yes
*R^2^*_adjust	0.7876	0.8129
*N*	7,829	7,829

Control variables include Lev, Growth, Bank, Roa, Mshare, Tq, Age, Balances, Board, Liqui, Pgdp, Awage. Subsequent tables are identical.

The symbols ***, **, and * respectively denote *p* < 0.01, *p* < 0.05, and *p* < 0.1.

### 5.3 Robust test

While the benchmark regression analysis has demonstrated that the reform of phased reduction of pension insurance contribution rate significantly influences the increase in the labor income share of enterprises, this paper undertakes a series of robustness tests to affirm the stability and reliability of the model’s estimation outcomes. We now examine the outcomes of select robustness tests, including the parallel trend assessment, propensity score matching method plus difference-in-differences method. For a comprehensive review of additional robustness tests, the Appendix is referred to.^12^

#### 5.3.1 Parallel trend test

Although the difference-in-differences method more effectively addresses endogeneity in model estimation, its application requires the fulfillment of a fundamental assumption: the sample of listed companies must adhere to the parallel trend assumption. This means that there should be no systematic differences in the labor income shares between the treatment and control groups of listed companies before the implementation of the reform policy of phased reduction of pension insurance contribution rate. Consequently, a parallel trend test is essential. This study extends the benchmark regression model to develop the following dynamic impact model for conducting the parallel trend test:


(11) $$\begin{array}{l} LS_{\mathrm{pit}}=\gamma_{0}+\overset{t=2018}{\underset{t=2014}{\sum }}\gamma_{t}Tt_{\mathrm{pi}}\times y_{t}+X_{\mathrm{pit}}\times \gamma \\ +Z_{\mathrm{pt}}\times \varpi +\mathrm{Fir}m_{i}+Year_{t}+\varphi_{\mathrm{pit}} \end{array}$$

where 2013 serves as the reference year, with $y_{t}$ representing a series of dummy variables for the years 2014–2018. Specifically, $y_{2014}$ equals 1 for the year 2014, $y_{2015}$ equals 1 for the year 2015, and this pattern continues for subsequent year. The term $\varphi_{\mathrm{pit}}$ denotes the random error term, while the remaining variables are defined as in Equation 10.

The result of the parallel trend test is presented in column (1) of Table 5. This test reveals that the regression coefficients for the interaction terms $Tt\times y_{2014}$ and $Tt\times y_{2015}$ are not statistically significant, indicating that prior to the implementation of the reform of phased reduction of pension insurance contribution rate, the labor income share of the treatment group did not exceed that of the control group in a statistically significant manner. Consequently, this supports the validity of the parallel trend assumption, a prerequisite for the difference-in-differences model.

**Table 5:** Parallel trend test, propensity score matching method plus difference-in-differences method.

Variable	(1)	(2)	(3)
	Parallel trend test	Propensity score matching method plus difference-in-differences method	
		Panel data transformation method	Period-by-period matching method
$Tt\times y_{2014}$	0.0039 (0.0040)		
$Tt\times y_{2015}$	0.0010 (0.0045)		
$Tt\times y_{2016}$	0.0075^*^ (0.0044)		
$Tt\times y_{2017}$	0.0091^*^ (0.0055)		
$Tt\times y_{2018}$	0.0103^**^ (0.0048)		
Tt×Pt		0.0072^***^ (0.0027)	0.0071*** (0.0027)
Constant	0.1319 (0.5036)	0.1421 (0.4917)	0.1449 (0.4911)
Controls	Yes	Yes	Yes
Firm /Year	Yes	Yes	Yes
*R^2^*_adjust	0.8100	0.8101	0.8102
*N*	7,833	7,830	7,826

The symbols ***, **, and * respectively denote *p* < 0.01, *p* < 0.05, and *p* < 0.1.

The coefficient estimates of the interaction terms $Tt\times y_{2016}$ , $Tt\times y_{2017}$ and $Tt\times y_{2018}$ are 0.0075, 0.0091, and 0.0103, respectively, and each is significantly positive at the 10% significance level or better. The increasing trend of the estimated coefficients of the individual interaction terms indicates that the positive impact of the reform of phased reduction of pension insurance contribution rate on the labor income share of enterprises has become progressively evident over a relatively brief period (2016–2018). This may stem from the fact that as the reform policy for the phased reduction of pension insurance contribution rate advances and the associated supporting policies continue to improve, the benefits of the reform are progressively realized. As a result, the reform’s effect on enhancing the labor income share within enterprises has been escalating.

#### 5.3.2 Propensity score matching method plus difference-in- differences method

An ideal scenario when using the difference-in-differences method to evaluate policy effects is that the individual characteristics of the treatment and control groups should be the same before the implementation of the reform policy. However, in practice, the diversity of regions where firms are situated and the inherent heterogeneity of the firms themselves result in significant differences in the enterprise characteristics between the treatment and control group samples. This variability can potentially introduce sample self-selection biases.

To mitigate the sample self-selection bias, this paper further uses propensity score matching method plus difference-in-differences method for robustness testing.

Considering that the propensity score matching method is suited for cross-sectional data and the difference-in-differences method is designed for panel data, this paper follows the approach outlined by Bai et al. (26). Initially, the panel data transformation method and the period-by-period matching approach are utilized to align propensity scores. Subsequently, using the newly formed samples post-matching, the difference-in-differences method is reapplied to assess the policy effects of the reform of phased reduction of pension insurance contribution rate.

The regression results based on these two methods are presented in columns (2) and (3) of Table 5. It is observed that the coefficient estimates of Tt×Pt are significantly positive at the 1% level, respectively. There is no significant alteration in the magnitude or significance of these coefficient estimates when compared to the benchmark regression results, suggesting that the findings from the benchmark regression analysis presented in this paper are robust.

## 6 Heterogeneity analysis and mechanism of action analysis

Prior empirical research, analyzing the causal effects from a comprehensive sample perspective, has already produced more robust findings. Nonetheless, it is yet to be determined whether the positive influence of the reform on the phased reduction of pension insurance contribution rate on labor income share varies due to differences in provincial location and ownership structure. To address this gap, the paper explores the following two dimensions of heterogeneity.

### 6.1 Heterogeneity analysis

#### 6.1.1 Heterogeneity analysis based on region

Variations in regional economic development levels lead to disparities in the potential for reducing pension insurance premiums across different regions. These disparities can significantly impact the effectiveness of the reform of phased reduction on the labor income share of enterprises. This paper divides the entire sample into two categories of enterprises in the eastern, central and western regions^13^ based on the province where the enterprise is registered, to investigate the differential impact of the reform on the labor income share of enterprises in different regions.

The regionally heterogeneous impact effects of the reform on the phased reduction of pension insurance contribution rate is presented in columns (1) and (2) of Table 6.

**Table 6:** Heterogeneity analysis.

Variable	(1)	(2)	(3)	(4)
	Eastern region	Central and western region	State-owned enterprises	Non-state-owned enterprises
Tt×Pt	0.0099^***^ (0.0031)	0.0074 (0.0071)	0.0102 (0.0125)	0.0071^**^ (0.0031)
Constant	0.2592 (0.6558)	0.0482 (0.7083)	0.0712 (1.9128)	−0.2449 (0.5585)
Controls	Yes	Yes	Yes	Yes
Firm/Year	Yes	Yes	Yes	Yes
*R*^2^_adjust	0.8199	0.7969	0.8191	0.8147
*N*	5,288	2,545	893	6,683

The symbols ***, **, and * respectively denote *p* < 0.01, *p* < 0.05, and *p* < 0.1.

The results show that among enterprises in the eastern region, the estimated coefficient of Tt×Pt is 0.0099 and significant at the 1% significance level, indicating that the reform of phased reduction of pension insurance contribution rate has a positive effect on increasing the labor income share of enterprises in the eastern region. Conversely, among enterprises in the central and western regions, the estimated coefficient of Tt×Pt , while positive, is not statistically significant. This indicates that the reform of phased reduction of pension insurance contribution rate does not significantly enhance the labor income share of enterprises in these regions.

The potential rationale for this regional differentiation lies in the eastern region’s higher economic development, stronger financial resources, and larger pension fund surplus compared to the central and western regions. These factors provide the eastern region with greater leeway to lower pension insurance premiums, thereby enhancing competitiveness (27). Consequently, the actual pension insurance contribution rate for enterprises in the eastern region is likely to decrease more substantially. Furthermore, the eastern region’s labor abundance means that reduced labor costs can significantly boost enterprise labor demand, leading to a more pronounced substitution effect of labor for capital. Thus, the reform’s policy effects exhibit regional heterogeneity.

#### 6.1.2 Heterogeneity analysis based on ownership

State-owned enterprises demonstrate a higher rate of compliance with pension insurance contributions, leading to a relatively higher pension insurance burden when compared with non-state-owned enterprises. This suggests that the structure of corporate ownership may significantly affect the effectiveness of the reform of phased reduction on the labor income share of enterprises. The differential impacts of this reform on *ownership*^14^ are detailed in columns (3) and (4) of Table 6.

The results show that the estimated coefficient of Tt×Pt in the sample of state-owned enterprises is 0.0102 and does not statistical significance. In contrast, the estimated coefficient of Tt×Pt is 0.0071 in the sample of non-state-owned enterprises and is significant at the 5% level. This suggests that the reform’s positive impact on the labor income share is more pronounced in non-state-owned enterprises compared to state-owned enterprises.

Possible explanations are that state-owned enterprises bear greater social responsibilities and political constraints, which may hinder their ability to adjust employment scales and employee wages. Consequently, the reform of pension insurance contribution rate has had limited influence on the labor income share of state-owned enterprises. In contrast, non-state-owned enterprises face heightened operational and competitive pressures and are more responsive to labor cost fluctuations. Their more flexible recruitment and wage mechanisms enable non-state-owned enterprises to rapidly adjust their allocation of production factors. Thus, the reform of phased reduction of pension insurance contribution rate has a greater effect on the actual pension insurance contribution rate of non-state-owned enterprises (27). Therefore, the reform of phased reduction of pension insurance contribution rate has a greater and more significant impact on the labor income share of non-state-owned enterprises, and does not have a significant impact on the labor income share of state-owned enterprises.

### 6.2 Mechanism of action analysis

The results of the empirical analysis in the previous section indicate that the reform of phased reduction of pension insurance contribution rate has a significant positive promotion effect on the labor income share of enterprises. Building on these findings, this paper will delve further into the underlying mechanisms.

Equation 11 indicates that the reform of phased reduction of pension insurance contribution rate promotes the increase of labor income share of enterprises by reducing the actual contribution rate of pension insurance and capital intensity. The change of pension insurance contribution rate caused by the reform of phased reduction of pension insurance contribution rate will change the actual pension insurance contribution rate of enterprises, and the change of the actual pension insurance contribution rate will affect the optimal combination of factor inputs and capital intensity of enterprises, which will affect the labor income share of enterprises. In summary, this paper argues that the reform of phased reduction of pension insurance contribution rate has a positive effect on the labor income share of enterprises by reducing the actual pension insurance contribution rate and the capital intensity. To this end, this paper constructs the following econometric model to verify:


(12) $$\begin{array}{l} \mathrm{Me}d_{\mathrm{pit}}=\mu_{0}+\mu_{1}Tt_{\mathrm{pi}}\times Pt_{\mathrm{pt}}+X_{\mathrm{pit}}\\ \;\times \xi +Z_{\mathrm{pt}}\times \pi +\mathrm{Fir}m_{i}+Year_{t}+\omega_{\mathrm{pit}} \end{array}$$

Where the dependent variable (*Med*) in Equation 12 represents the actual contribution rate of pension insurance (*Rate*) and the capital intensity (*KL*). The other variables retain the same definitions as in Equation 10.

The outcomes of the mechanism analysis for the reform of phased reduction of the pension insurance contribution rate affecting the labor income share of enterprises are shown in Table 7. In column (1), the independent variable is *Rate*, which is expressed as the ratio of the current increase in pension insurance to (“current increase in payable employees’ compensation” – “directors’ and supervisors’ compensation”). The estimated coefficient of Tt×Pt is −0.0044, significantly negative at the 1% level, indicating that the reform of phased reduction of pension insurance contribution rate has a significant inhibitory effect on the actual pension insurance contribution rate of enterprises. In column (2), the dependent variable is *KL*, defined as the natural logarithm of net fixed assets per capita. The estimated coefficient of Tt×Pt is −0.0412, significantly negative at the 10% level, indicating that the reform of phased reduction of pension contribution rate has a significant dampening effect on capital intensity.

**Table 7:** Mechanism of action tests.

Variable	(1)	(2)
	*Rate*	*KL*
Tt×Pt	−0.0044*** (0.0007)	−0.0412* (0.0224)
Constant	0.1244 (0.1231)	19.4487*** (3.0553)
Controls	Yes	Yes
Firm/Year	Yes	Yes
*R*^2^_adjust	0.8155	0.8605
*N*	7,507	7,828

The symbols ***, **, and * respectively denote *p* < 0.01, *p* < 0.05, and *p* < 0.1.

The regression analysis indicates that the reform of phased reduction of pension insurance contribution rate has led to a decrease in the actual contribution rate of enterprises pension insurance. A reduced actual contribution rate of pension insurance implies lower labor costs, which encourages enterprises to adjust their production input decisions by substituting labor for capital, thereby reducing capital intensity and consequently promoting an increase in the labor income share. In conclusion, the actual contribution rate of pension insurance and the capital intensity are two possible mechanisms through which the reform of phased reduction of pension insurance contribution rate affects the labor income share of enterprises^15^.

## 7 Conclusion and policy recommendations

Utilizing the 2016 reform of the phased reduction of pension insurance contribution rate as a quasi-natural experiment, this paper empirically investigates the impact of this reform on the labor income share of enterprises and elucidates its operational mechanisms. The analysis uses a difference-in-differences model, leveraging data from China’s A-share listed companies spanning 2013–2018. Subsequently, robustness checks are performed using diverse methods and heterogeneity analyses based on both regional and ownership factors. Additionally, the study examines the reform’s operational mechanism in enhancing the labor income share of enterprises, focusing on the actual pension insurance contribution rate and capital intensity.

The results of this paper are as follows:

1. The reform of phased reduction of pension insurance contribution rate positively influences the labor income share of enterprises.
2. Heterogeneity analysis based on both region and ownership factors reveal that the phased reduction of pension insurance contribution rate is more likely to significantly contribute to the increase in the labor income share of enterprises in the eastern region and in non-state-owned enterprises.
3. The analysis of the mechanism of action suggests that the actual pension insurance contribution rate and the capital intensity are two possible mechanisms through which the reform of phased reduction of pension insurance contribution rate affects the labor income share of enterprises.

Based on the above research findings, this paper offers the following policy recommendations:

1. This paper proposes a reduction in the pension insurance contribution rate, complemented by enhanced pension insurance management. Relative to other developed nations, China’s mandatory corporate pension insurance contribution rate is considerably higher, suggesting ample scope for a reduction. Based on the prevailing conditions, it is feasible to further lower the pension insurance contribution rate. Concurrently, it is imperative to reinforce the management of pension insurance to mitigate potential negative impacts on the revenue of pension insurance funds. This approach aims to harmonize the implementation of “rate reduction” with “enhanced collection and management” strategies.
2. Policies should be tailored to account for regional disparities and ownership variations, thereby enhancing the efficacy of the pension insurance contribution reduction reform. For central and western regions, strategies such as pension insurance contribution subsidies or alternative tax incentives could be implemented to counteract the rigidity of pension insurance contribution regulations and bolster the effectiveness of the policy. For state-owned enterprises, developing a flexible wage adjustment system and recruitment mechanisms will enable enterprises to adapt their factor inputs accordingly.

## Data Availability

The raw data supporting the conclusions of this article will be made available by the authors, without undue reservation.

## Footnotes

^1^The China Statistical Yearbook after 2018 no longer publishes the items that make up the income approach to regional GDP, while relying on Bai et al. (26) and Lan et al. (28) on the measurement of labor income share. The labor income share is calculated for 2000–2017.

^2^Pension insurance contribution rate in paper refer to the statutory pension insurance contribution rate.

^3^Source: Pension insurance policy documents published by the provinces.

^4^Data source: http://www.mohrss.gov.cn/sybxs/SYBXSzhengcewenjian/SYBXSguojiazhengce/201804/t20180420_292714.html.

^5^Includes Beijing, Tianjin, Chongqing, Sichuan, Anhui, Jiangxi, Xinjiang, Shanxi, Henan, Hubei, Guangxi, Guizhou, Hunan, Gansu, Ningxia, Jiangsu, Hainan and Yunnan.

^6^Includes Guangdong, Hebei, Fujian, Zhejiang, Liaoning, Jilin, Qinghai, Tibet, Shandong, Inner Mongolia and Shaanxi.

^7^Data source: Pensions at a Glance 2017.

^8^This paper also empirically tests the factor elasticity of substitution between capital and labor using relevant data. A regression of the labor income share on capital intensification reveals that the estimated coefficient on capital intensification is significantly negative, suggesting a substitution relationship between capital and labor.

^9^The theoretical derivation of the relationship between capital intensity and the actual contribution rate of pension insurance is presented in the Appendix.

^10^2.02% = 0.0058/0.2871, where 0.2871 is the average of the labor income share.

^11^2.51% = 0.0072/0.2871, where 0.2871 is the average of the labor income share.

^12^Other robustness test results include dependent variables replacement, placebo test, exclusion of other policy interferences, time grouping reset and heterogeneity processing effect.

^13^The eastern region includes Beijing, Tianjin, Hebei, Liaoning, Shanghai, Jiangsu, Zhejiang, Fujian, Shandong, Guangdong, and Hainan; the central and western region includes Shanxi, Jilin, Heilongjiang, Anhui, Jiangxi, Henan, Hubei, Hunan, Inner Mongolia, Guangxi, Chongqing, Sichuan, Guizhou, Yunnan, Shaanxi, Gansu, Qinghai, Ningxia, and Xinjiang.

^14^Ownership is a dummy variable. When the nature of the shares for the state, state-owned legal persons, state-owned legal persons in the territory of the state-owned enterprises, Ownership takes the value of 1, other non-state-owned enterprises, Ownership takes the value of 0; controlling shareholders if there are more than one, as long as one of them is a state-owned enterprise, Ownership takes the value of 1.

^15^To enhance the reliability of our findings, we conducted a Sobel test following the methodology outlined by Wei and Xia (29). The results confirmed the validity of our conclusions.
