# Supplementary material for: Does the reform of the phased reduction of the pension insurance contribution rate benefit the labor income share of enterprises?
Source: Front Public Health. 2025 Jan 17;12:1473166. doi: 10.3389/fpubh.2024.1473166 (PMC11782555; doi:10.3389/fpubh.2024.1473166)
Supplement: Supplementary file 1 [file Table_1.docx]

# Appendix

## **1 Other derivation processes of theoretical model**

The theoretical derivation of the relationship between capital intensity and the actual pension insurance contribution rate is as follows: By substituting equation (7) into equation (3) and rearranging the terms, it can be obtained:

$k_{i}\left( \tau_{i} \right)=\left( 1+\theta\tau_{i} \right)^{\sigma}M^{\sigma}\left( \frac{w_{i}}{r_{i}} \right)^{\sigma}$ （13）

Equation (13) explicitly establishes the relationship between the actual pension insurance contribution rate and capital intensity. Consequently, the partial derivative of capital intensity ($k_{i}$) with respect to the actual pension insurance contribution rate ($\tau_{i}$) can be derived from Equation:

$\frac{\partial k_{i}\left( \tau_{i} \right)}{\partial\tau_{i}}=\left( \frac{\alpha}{1-\alpha} \right)^{\sigma}\left( \frac{w_{i}}{r_{i}} \right)^{\sigma}\theta\sigma\left( 1+\theta\tau_{i} \right)^{\sigma-1}$ （14）

It is evident that Equation (14) is consistently positive. Hence, an increase in the actual pension insurance contribution rate leads to an increase in a firm's capital intensity, implying that $\frac{\partial k_{i}\left( \tau_{i} \right)}{\partial\tau_{i}}$ is greater than 0.

## **2 Mean description and t-test**

This paper categorizes all listed companies into treatment and control group samples and conducts group statistics. Table A reports the mean and t-test of labor income shares of listed companies in both groups. The mean labor income share of companies in the treatment group exceeds that of the control group, with a significant difference at the 5% level. This suggests a notable disparity in labor income shares between the treatment and control groups. Collectively, the mean t-test results provide preliminary evidence that the reform of the phased reduction in pension insurance contribution rate significantly enhances the labor income share of enterprises.

TABLE A Mean description and t-test of labor income share of listed companies in the treatment and control groups

| Variable | Treatment Group | Control Group | Mean Difference |
| --- | --- | --- | --- |
| *LS* | 0.2899 | 0.2836 | 0.0063^**^ |

## **3 Other robust tests**

### **3.1 Dependent variable replacement**

This paper conducts a robustness test to ascertain the reliability of the empirical estimation results by substituting alternative measures for the dependent variable, thereby mitigating potential biases stemming from the measurement of labor income share in the benchmark regression analysis.

Labor compensation is measured by "cash paid to and for employees" and then the labor income share (*LS1*) equal to "labor compensation " divided by "value added of the enterprise"; Labor compensation is measured by "cash paid to and for employees + compensation payable at the end of the period - compensation payable at the beginning of the period", and then the labor income share (*LS2*) equal to "labor compensation" divided by "total assets of the enterprise"; Referring to the study of Zhu et al. (2022), labor compensation is measured by "Cash paid to and for employees + Employee compensation payable at the end of the period of the enterprise - Employee compensation payable at the beginning of the period of the enterprise", and then the labor income share (*LS3*) equal to "labor compensation" divided by "value added of the enterprise"; Referring to the study of Wang and Huang (2017), labor compensation is measured by "credit incidence of employee compensation payable", and then the labor income share (*LS4*) is equal to "labor compensation" divided by "total business revenue of the firm"; Since the labor income share takes values in the range of (0, 1), which does not conform to a normal distribution, the labor income share (LS5) is calculated by taking the natural logarithm of the labor income share (*LS*), adjusted to LS/(1-LS) by logistic transformation.

Table B presents the estimation results after replacing the dependent variable. The regression analyses depicted in columns (1) through (5) reveal that the coefficients for the independent variables $Tt\times Pt$ remain significantly positive at the 5% significance level.

Variations in these coefficients are confined to their magnitude and the level of statistical significance. Collectively, these findings indicate that the reform of the phased reduction of pension insurance contribution rate is beneficial for enhancing the labor income share of enterprises, thereby reinforcing the robustness of the benchmark regression results presented in this paper.

TABLE B Dependent variable replacement

| Variable | （1） | （2） | （3） | （4） | （5） |
| --- | --- | --- | --- | --- | --- |
|  | *LS1* | *LS2* | *LS3* | *LS4* | *LS5* |
| $Tt\times Pt$ | 0.0055^**^(0.0025) | 0.0017^**^(0.0007) | 0.0059^**^(0.0025) | 0.0050^***^(0.0015) | 0.0346^**^(0.0138) |
| *Constant* | 0.0683(0.4702) | -0.0987(0.1190) | 0.0111(0.4563) | 0.1387(0.2483) | -2.2598(2.6471) |
| *Controls* | Yes | Yes | Yes | Yes | Yes |
| *Firm/Year* | Yes | Yes | Yes | Yes | Yes |
| *R^2^*_adjust | 0.8125 | 0.8809 | 0.8105 | 0.8497 | 0.8223 |
| *N* | 7833 | 7778 | 7778 | 7833 | 7833 |

The symbols *** and ** respectively denote p<0.01 and p<0.05.

### **3.2 Placebo test**

To bolster the robustness of the findings, two sequential placebo tests were conducted:

i) A sham treatment and control group were established via random sampling. Specifically, treatment and control groups were randomly assigned, with 20 out of 30 provinces selected at random. The sample of listed companies domiciled in these selected provinces constituted the sham treatment group, while those domiciled in the remaining provinces formed the sham control group. The baseline regression model was applied to these groups, with the process repeated 500 times. The regression outcomes for the independent variable were documented. As detailed in Table C the statistical distribution of the sham treatment effects shows that the percentages of coefficients significantly positive or negative are minimal, and the mean coefficient value is 0.0001, nearly zero, suggesting that the constructed sham treatment effect is negligible. This, in turn, corroborates the reliability and robustness of the study's findings.

TABLE C Statistical distribution of regression results for spurious treatment effect

|  | *N* | Mean | Std | p5 | p25 | p50 | p75 | p95 |
| --- | --- | --- | --- | --- | --- | --- | --- | --- |
| Coefficient | 500 | 0.0001 | 0.0043 | -0.0075 | -0.0028 | 0.0002 | 0.0028 | 0.0068 |
| T-value | 500 | 0.0003 | 1.4643 | -2.5156 | -0.9685 | 0.0502 | 0.9645 | 2.3465 |

ii) Replacement of the spurious policy implementation date. By advancing the implementation year of the reform of phased reduction of pension insurance contribution rate by one year and two years, respectively, and taking 2015 or 2014 as the implementation year of the reform policy, thus constructing the spurious independent variable and regressing them using the benchmark regression model, and the specific results are shown in columns (1) and (2) of Table D. The estimated coefficients of the spurious independent variable $Tt\times Pre1$ and $Tt\times Pre2$ are both insignificantly positive, which is not consistent with the results of the benchmark regression in this paper, implying that the spurious treatment effect does not hold. Both of these placebo test results again demonstrate the robustness of the benchmark regression results in this paper.

### **3.3 Exclusion of other policy interferences**

The accuracy of the model’s estimation results may also be affected if other policy shocks occurred during the 2013-2018 period and if these policy shocks had an impact on enterprises’ labor income share. This paper finds that the accelerated depreciation policy for fixed assets implemented in 2014 may have interfered with the results of this paper. This policy allows enterprises in six key industries^[[1]](#footnote-1)^, including the biopharmaceutical manufacturing industry, to accelerate the depreciation of newly acquired fixed assets (Xu et al., 2021), which reduces the relative price of capital, which in turn may have an impact on the labor income share. For this reason, this paper adopts the following two methods for robustness testing: i) Replace the time fixed effects in the benchmark regression model with industry-time interaction fixed effects to control for disturbances caused by time-varying policies in different industries. ii) After deleting the samples of listed companies in six key industries, such as biological drug manufacturing, the benchmark regression model is regressed again. The specific regression results are shown in columns (3) and (4) of Table D. It can be seen that the estimated coefficients of the independent variable $Tt\times Pt$ are still significantly positive after excluding the effect of the accelerated depreciation policy on fixed assets, proving once again that the reform of phased reduction of pension insurance contribution rate is indeed conducive to the increase of labor income share of enterprises in the provinces where the reform is implemented, and that the conclusions of this paper are robust.

### **3.4 Time grouping reset**

In the main text, there may be a problem of incorrectly setting the variables when setting the dummy variables for time grouping. In fact, the reform of phased reduction of pension insurance contribution rate in all provinces has been implemented since May 2016, and it may be inaccurate to set the $Pt$ in 2016 as 1 in this paper. Therefore, here we set $Pt$ in 2016 as 2/3^[[2]](#footnote-2)^, and $Pt$ in 2017-2018 as 1, and then regress equation 10, and the relevant regression results are shown in column (5) of Table D. It can be found that the estimated coefficients of the independent variable $Tt\times Pt$ are still significantly positive at the 1% level, indicating that the reform of phased reduction of pension insurance contribution rate is conducive to the improvement of the labor income share of enterprises, which once again proves that the research conclusions of this paper are robust.

TABLE D Placebo test, exclusion of other policy interferences, time grouping reset

| Variable | (1) | (2) | （3） | （4） | （5） |
| --- | --- | --- | --- | --- | --- |
|  | Placebo test | | Exclusion of other policy interferences | | Time grouping reset |
|  | 1 year ahead | 2 years ahead | i | ii |  |
| $Tt\times Pt$ |  |  | 0.0083^***^(0.0026) | 0.0056^*^(0.0032) |  |
| $Tt\times Pt1$ |  |  |  |  | 0.0072^***^(0.0027) |
| $Tt\times Pre1$ | 0.0043(0.0028) |  |  |  |  |
| $Tt\times Pre2$ |  | 0.0056(0.0039) |  |  |  |
| Constant | 0.2135(0.4832) | 0.1985(0.4951) | 0.2812(0.4735) | 0.4818(0.6155) | 0.1593(0.4905) |
| *Controls* | Yes | Yes | Yes | Yes | Yes |
| *Firm* | Yes | Yes | Yes | Yes | Yes |
| *Year* | Yes | Yes | No | Yes | Yes |
| *Industry-time* | No | No | Yes | No | No |
| *R^2^*_adjust | 0.8100 | 0.8100 | 0.8253 | 0.8035 | 0.8101 |
| *N* | 7833 | 7833 | 7790 | 5713 | 7833 |

Industry-time indicate industry-time interaction fixed effects. The symbols *** and * respectively denote p<0.01 and p<0.1.

3.5 Heterogeneity processing effect

Considering that the estimation results of traditional bidirectional fixed effects models may be biased, this paper uses the twowayfeweights command to conduct robustness tests on the possible heterogeneity treatment effects of the model. The analysis reveals that all 1972 weights are positive, suggesting that heterogeneity in treatment effects exerts a negligible influence on the estimation outcomes. Consequently, the benchmark regression results presented in this paper are deemed robust.

1. The six key industries specifically include the biopharmaceutical manufacturing industry, the railway, ship, aerospace and other transport equipment manufacturing industry, the special-purpose equipment manufacturing industry, the information transmission, software and information technology services industry, the computer, communications and other electronic equipment manufacturing industry, and the instrument and meter manufacturing industry. [↑](#footnote-ref-1)
2. In fact, the reform of the phased reduction of the pension contribution rate was implemented from May 2016 to the end of December 2016, which is only eight months, accounting for 8/12 = 2/3 of the year. [↑](#footnote-ref-2)
